# *N*,*N*-Bis(quinolin-8-yl)-2,2′-[(1,3,4-thia­diazole-2,5-di­yl)bis­(sulfanedi­yl)]diacetamide monohydrate

**DOI:** 10.1107/S1600536811047222

**Published:** 2011-11-12

**Authors:** Xiao-Feng Li, Yan An, Qing-Hua Huang, Yong-Hong Wen

**Affiliations:** a Institute of Marine Materials Science and Engineering, Shanghai Maritime University, Shanghai 201305, People’s Republic of China; b College of Chemistry and Molecular Engineering, Qingdao University of Science and Technology, Qingdao 266042, People’s Republic of China

## Abstract

In the title compound, C_24_H_18_N_6_O_2_S_3_·H_2_O, the thia­diazole ring makes dihedral angles of 78.00 (13) and 77.27 (13)° with the quinoline ring systems. In the crystal, mol­ecules are linked into a two-dimensional network by O—H⋯O and C—H⋯O hydrogen bonds.

## Related literature

For background to the applications of 2,5-dimercapto-1,3,4-thia­diazole, see: Vullo *et al.* (2003 ▶); Gurn (2001 ▶). For related 2,5-dimercapto-1,3,4-thia­diazole structures, see: Wen *et al.* (2005 ▶); Zhang *et al.* (2005 ▶).
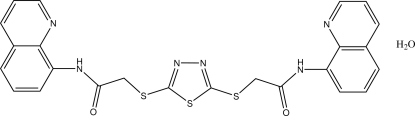


## Experimental

### 

#### Crystal data

C_24_H_18_N_6_O_2_S_3_·H_2_O
*M* *_r_* = 536.64
Monoclinic, 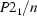

*a* = 10.8215 (8) Å
*b* = 10.2355 (8) Å
*c* = 21.5510 (16) Å
β = 90.068 (1)°
*V* = 2387.1 (3) Å^3^
*Z* = 4
Mo *K*α radiation
μ = 0.35 mm^−1^
*T* = 293 K
0.15 × 0.10 × 0.10 mm

#### Data collection

Bruker APEXII CCD area-detector diffractometer
Absorption correction: multi-scan (*SADABS*; Sheldrick, 1996 ▶) *T*_min_ = 0.949, *T*_max_ = 0.966
12711 measured reflections
4542 independent reflections
3469 reflections with *I* > 2σ(*I*)
*R*_int_ = 0.031

#### Refinement

*R*[*F* ^2^ > 2σ(*F* ^2^)] = 0.044
*wR*(*F* ^2^) = 0.109
*S* = 1.02
4542 reflections
333 parameters
3 restraints
H atoms treated by a mixture of independent and constrained refinement
Δρ_max_ = 0.28 e Å^−3^
Δρ_min_ = −0.19 e Å^−3^

### 

Data collection: *APEX2* (Bruker 2001 ▶); cell refinement: *SAINT* (Bruker 2001 ▶); data reduction: *SAINT*; program(s) used to solve structure: *SHELXTL* (Sheldrick, 2008 ▶); program(s) used to refine structure: *SHELXTL*; molecular graphics: *SHELXTL*; software used to prepare material for publication: *SHELXTL*.

## Supplementary Material

Crystal structure: contains datablock(s) I, global. DOI: 10.1107/S1600536811047222/hg5124sup1.cif

Structure factors: contains datablock(s) I. DOI: 10.1107/S1600536811047222/hg5124Isup2.hkl

Supplementary material file. DOI: 10.1107/S1600536811047222/hg5124Isup3.cml

Additional supplementary materials:  crystallographic information; 3D view; checkCIF report

## Figures and Tables

**Table 1:** Hydrogen-bond geometry (Å, °)

*D*—H⋯*A*	*D*—H	H⋯*A*	*D*⋯*A*	*D*—H⋯*A*
O1*W*—H1*WA*⋯O1	0.95 (7)	2.07 (7)	2.990 (3)	162 (6)
O1*W*—H1*WB*⋯O2^i^	0.94 (4)	1.91 (4)	2.841 (3)	175 (4)
C11—H11*B*⋯O1*W*^ii^	0.97	2.49	3.332 (4)	145
C23—H23⋯O1^iii^	0.93	2.55	3.411 (4)	155

Symmetry codes: (i) 
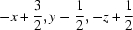
; (ii) 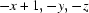; (iii) 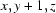.

**Fig. 1.**
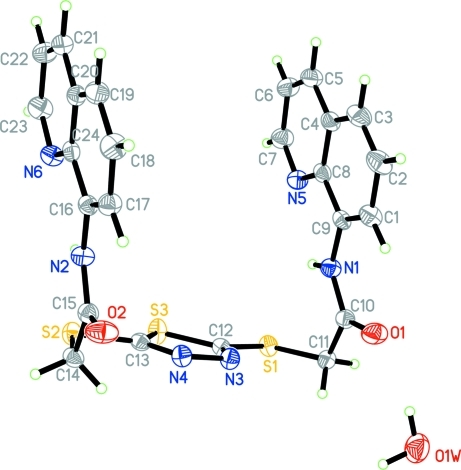
The structure of the title compound, showing the atom-labelling scheme. Displacement ellipsoids are drawn at the 30% probability level.

**Fig. 2.**
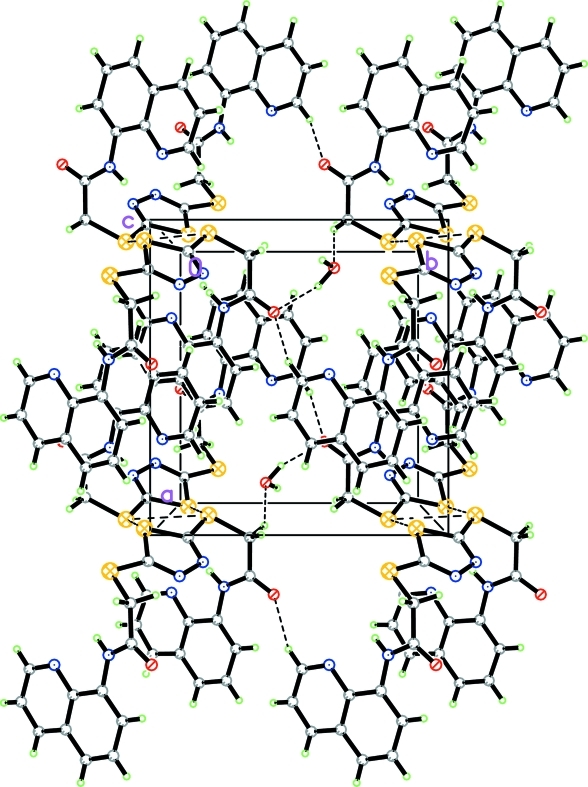
The packing diagram of the title compound, viewed down the c axis.

## supplementary crystallographic information

### Comment

Thiodiazole and its derivatives have attracted much attention in the development of the new kind pesticide. 2,5-Dimercapto-1,3,4-thiadiazole (DMTD) is an effective stabilizer for emulsions, and its derivatives can be absorbed by plant cells, so they can be prepared as bactericides, herbicides and insecticides, *etc* (Vullo *et al.*, 2003; Gurn, 2001). In this paper, a new DMTD derivative of amide-based open-chain crown-ether, 2,5-di(quinolin-8-ylcarbamoylmethylthio)-1,3,4-thiodiazole, was synthesized and an X-ray crystal structure undertaken to elucidate its molecular conformation (Fig. 1).

The crystal structure of the title compound, consists of a C_24_H_18_N_6_O_2_S_3_ molecule and a crystal water molecule. All bond lengths and angles in the title compound are within normal ranges, and are comparable with the structural related compounds (Wen *et al.*, 2005; Zhang *et al.*, 2005). The bond lengths in thiadiazole ring show a character intermediate between single and double bond because of the π-conjugation. The thiadiazole ring and two quinoline rings are each coplanar with their attached atoms, excluding the H atoms attached to them, while the whole molecule is not planar, with dihedral angles of 78.00 (13) and 77.27 (13)° between the thiadiazole ring and the two quinoline rings, respectively. The N1 atom adopts a planar configuration with the sum of the bond angles around atom N1 being 360.00°.

In the crystal packing, the molecules are linked into network structure by O1W—H1WA···O1, O1W—H1WB···O2, C11—H11B···O1W and C23—H23A···O1 hydrogen bond interactions (Table 1, Fig. 2).

### Experimental

After stirring the 40 ml acetone solution of 2,5-dimercapto-1,3,4-thiodiazole (1.50 g, 10 mmole), K_2_CO_3_ (1.52 g, 11 mmole) and NaI (0.5 g) at room temperature for 30 minutes, a 20 ml solution of 2-chloro-*N*-quinolin-8-ylacetamide (4.41 g, 20 mmoles) in acetone was added drop by drop, and the mixture was refluxed at 329 K for 3 h. After cooling to room temperature, the mixture was washed three times with water (3 × 5 ml) and then filtered. The filter cake was washed three times with acetone (3 × 5 ml). 2,5-di(quinolin-8-ylcarbamoylmethylthio)-1,3,4-thiodiazole was obtained after dryness of the resulting coffee powders at room temperature for 48 h. Yield 3.91 g (80.1%), mp 166.5–167.5°C. Anal. Calcd. (%) for C_24_H_18_N_6_O_2_S_3_: C, 55.58; H, 3.50; N, 16.20; S, 18.55. Found (%): C, 55.62; H, 3.55; N, 16.26; S, 18.53.

Brown single crystals suitable for X-ray diffraction analysis were obtained by slow evaporation at room temperature from CH_3_CH_2_OH for 15 days.

### Refinement

Water molecule bound H atoms were located in difference Fourier maps and their positional parameters refined with a distance restraint [O—H = 0.85 (10) Å] and a angle restraint. Other H atoms were positioned geometrically, with N—H = 0.86 Å and C—H = 0.95–0.99 Å, respectively, and constrained to ride on their parent atoms, with *U*_iso_(H) = 1.2 *U*_eq_(C,*N*).

### Crystal data

**Table:**

C_24_H_18_N_6_O_2_S_3_·H_2_O	*F*(000) = 1112
*M**_r_* = 536.64	*D*_x_ = 1.493 Mg m^−^^3^
Monoclinic, *P*2_1_/*n*	Mo *K*α radiation, λ = 0.71073 Å
Hall symbol: -P 2yn	Cell parameters from 2913 reflections
*a* = 10.8215 (8) Å	θ = 2.2–24.6°
*b* = 10.2355 (8) Å	µ = 0.35 mm^−^^1^
*c* = 21.5510 (16) Å	*T* = 293 K
β = 90.068 (1)°	Prism, brown
*V* = 2387.1 (3) Å^3^	0.15 × 0.10 × 0.10 mm
*Z* = 4	

### Data collection

**Table:**

Bruker APEXII CCD area-detector diffractometer	4542 independent reflections
Radiation source: fine-focus sealed tube	3469 reflections with *I* > 2σ(*I*)
graphite	*R*_int_ = 0.031
phi and ω scans	θ_max_ = 25.7°, θ_min_ = 1.9°
Absorption correction: multi-scan (*SADABS*; Sheldrick, 1996)	*h* = −11→13
*T*_min_ = 0.949, *T*_max_ = 0.966	*k* = −12→11
12711 measured reflections	*l* = −26→26

### Refinement

**Table:**

Refinement on *F*^2^	Primary atom site location: structure-invariant direct methods
Least-squares matrix: full	Secondary atom site location: difference Fourier map
*R*[*F*^2^ > 2σ(*F*^2^)] = 0.044	Hydrogen site location: inferred from neighbouring sites
*wR*(*F*^2^) = 0.109	H atoms treated by a mixture of independent and constrained refinement
*S* = 1.02	*w* = 1/[σ^2^(*F*_o_^2^) + (0.0551*P*)^2^ + 0.3494*P*] where *P* = (*F*_o_^2^ + 2*F*_c_^2^)/3
4542 reflections	(Δ/σ)_max_ < 0.001
333 parameters	Δρ_max_ = 0.28 e Å^−^^3^
3 restraints	Δρ_min_ = −0.19 e Å^−^^3^

### Special details

**Table:**

Geometry. All e.s.d.'s (except the e.s.d. in the dihedral angle between two l.s. planes) are estimated using the full covariance matrix. The cell e.s.d.'s are taken into account individually in the estimation of e.s.d.'s in distances, angles and torsion angles; correlations between e.s.d.'s in cell parameters are only used when they are defined by crystal symmetry. An approximate (isotropic) treatment of cell e.s.d.'s is used for estimating e.s.d.'s involving l.s. planes.
Refinement. Refinement of *F*^2^ against ALL reflections. The weighted *R*-factor *wR* and goodness of fit *S* are based on *F*^2^, conventional *R*-factors *R* are based on *F*, with *F* set to zero for negative *F*^2^. The threshold expression of *F*^2^ > σ(*F*^2^) is used only for calculating *R*-factors(gt) *etc*. and is not relevant to the choice of reflections for refinement. *R*-factors based on *F*^2^ are statistically about twice as large as those based on *F*, and *R*- factors based on ALL data will be even larger.

### Fractional atomic coordinates and isotropic or equivalent isotropic displacement parameters (Å^2^)

**Table:**

	*x*	*y*	*z*	*U*_iso_*/*U*_eq_	
S1	0.48854 (6)	0.33999 (6)	0.03568 (3)	0.04181 (18)	
S2	0.65584 (6)	0.67713 (6)	0.23342 (3)	0.04294 (18)	
S3	0.52541 (6)	0.56902 (7)	0.12152 (3)	0.0484 (2)	
N2	0.93916 (17)	0.68189 (19)	0.23701 (8)	0.0376 (5)	
H2	0.8860	0.7272	0.2166	0.045*	
N4	0.68075 (18)	0.4337 (2)	0.18382 (9)	0.0409 (5)	
N5	0.78366 (18)	0.4925 (2)	−0.08496 (9)	0.0445 (5)	
N6	0.98890 (17)	0.8914 (2)	0.16646 (9)	0.0422 (5)	
O2	0.95579 (16)	0.5195 (2)	0.30789 (9)	0.0598 (5)	
C24	1.0876 (2)	0.8188 (2)	0.18599 (10)	0.0342 (5)	
C16	1.0642 (2)	0.7078 (2)	0.22398 (10)	0.0344 (5)	
O1	0.76858 (17)	0.09593 (19)	0.04223 (9)	0.0592 (5)	
C8	0.8872 (2)	0.4250 (2)	−0.06819 (10)	0.0375 (5)	
C10	0.7060 (2)	0.1861 (2)	0.02251 (10)	0.0395 (6)	
C20	1.2107 (2)	0.8493 (2)	0.16943 (11)	0.0374 (5)	
C15	0.8927 (2)	0.5951 (2)	0.27744 (10)	0.0383 (5)	
C9	0.8725 (2)	0.3130 (2)	−0.02972 (10)	0.0382 (5)	
N3	0.63967 (18)	0.35305 (19)	0.13605 (8)	0.0413 (5)	
N1	0.75026 (17)	0.2858 (2)	−0.01150 (9)	0.0416 (5)	
H1	0.6958	0.3411	−0.0240	0.050*	
C13	0.62927 (19)	0.5483 (2)	0.18185 (10)	0.0353 (5)	
C4	1.0072 (2)	0.4606 (3)	−0.08780 (11)	0.0435 (6)	
C12	0.5590 (2)	0.4104 (2)	0.10073 (10)	0.0361 (5)	
C14	0.7546 (2)	0.5972 (2)	0.28903 (10)	0.0398 (6)	
H14A	0.7410	0.6384	0.3290	0.080*	
H14B	0.7271	0.5074	0.2927	0.080*	
C11	0.5678 (2)	0.1851 (2)	0.03428 (11)	0.0416 (6)	
H11A	0.5537	0.1423	0.0738	0.080*	
H11B	0.5295	0.1314	0.0025	0.080*	
C1	0.9732 (2)	0.2404 (3)	−0.01294 (12)	0.0499 (7)	
H1A	0.9633	0.1663	0.0115	0.060*	
C19	1.3081 (2)	0.7714 (3)	0.19269 (12)	0.0467 (6)	
H19	1.3895	0.7920	0.1831	0.056*	
C21	1.2295 (2)	0.9559 (3)	0.12920 (12)	0.0461 (6)	
H21	1.3089	0.9783	0.1166	0.055*	
C22	1.1310 (3)	1.0255 (3)	0.10913 (12)	0.0513 (7)	
H22	1.1418	1.0958	0.0824	0.062*	
C6	0.9135 (3)	0.6360 (3)	−0.14421 (12)	0.0560 (7)	
H6	0.9186	0.7077	−0.1705	0.067*	
C17	1.1607 (2)	0.6324 (2)	0.24427 (11)	0.0432 (6)	
H17	1.1456	0.5584	0.2682	0.052*	
C18	1.2827 (2)	0.6669 (3)	0.22894 (12)	0.0506 (7)	
H18	1.3476	0.6166	0.2441	0.061*	
C2	1.0919 (2)	0.2781 (3)	−0.03267 (13)	0.0585 (8)	
H2A	1.1599	0.2284	−0.0208	0.070*	
C23	1.0124 (3)	0.9901 (3)	0.12920 (12)	0.0517 (7)	
H23	0.9459	1.0397	0.1153	0.062*	
C7	0.7990 (3)	0.5934 (3)	−0.12173 (12)	0.0542 (7)	
H7	0.7290	0.6398	−0.1337	0.065*	
C3	1.1092 (2)	0.3847 (3)	−0.06834 (13)	0.0552 (7)	
H3	1.1887	0.4084	−0.0802	0.066*	
C5	1.0168 (3)	0.5704 (3)	−0.12682 (12)	0.0520 (7)	
H5	1.0939	0.5980	−0.1407	0.062*	
O1W	0.6164 (3)	−0.1290 (2)	0.08683 (12)	0.0789 (7)	
H1WA	0.679 (6)	−0.071 (7)	0.073 (3)	0.27 (4)*	
H1WB	0.593 (4)	−0.085 (4)	0.1230 (19)	0.130 (17)*	

### Atomic displacement parameters (Å^2^)

**Table:**

	*U*^11^	*U*^22^	*U*^33^	*U*^12^	*U*^13^	*U*^23^
S1	0.0407 (4)	0.0475 (4)	0.0372 (3)	0.0041 (3)	−0.0073 (3)	−0.0041 (3)
S2	0.0381 (3)	0.0438 (4)	0.0470 (4)	0.0016 (3)	−0.0049 (3)	−0.0076 (3)
S3	0.0509 (4)	0.0457 (4)	0.0487 (4)	0.0147 (3)	−0.0176 (3)	−0.0088 (3)
N2	0.0308 (10)	0.0446 (12)	0.0373 (10)	0.0012 (8)	−0.0025 (8)	0.0077 (9)
N4	0.0423 (11)	0.0442 (13)	0.0363 (11)	0.0041 (9)	−0.0060 (9)	−0.0003 (9)
N5	0.0432 (12)	0.0470 (13)	0.0434 (12)	−0.0013 (10)	0.0001 (9)	0.0033 (10)
N6	0.0392 (11)	0.0471 (13)	0.0403 (11)	0.0049 (9)	0.0019 (9)	0.0064 (10)
O2	0.0464 (11)	0.0685 (13)	0.0645 (12)	0.0073 (9)	0.0055 (9)	0.0286 (10)
C24	0.0353 (13)	0.0353 (13)	0.0319 (11)	0.0013 (10)	−0.0001 (10)	−0.0070 (10)
C16	0.0321 (12)	0.0392 (14)	0.0321 (12)	0.0000 (10)	−0.0004 (9)	−0.0036 (10)
O1	0.0534 (11)	0.0576 (13)	0.0666 (12)	0.0114 (9)	0.0089 (9)	0.0233 (10)
C8	0.0389 (13)	0.0430 (14)	0.0307 (12)	−0.0013 (10)	0.0004 (10)	−0.0067 (11)
C10	0.0451 (14)	0.0397 (14)	0.0337 (12)	0.0017 (11)	−0.0002 (10)	0.0017 (11)
C20	0.0350 (13)	0.0375 (14)	0.0398 (13)	−0.0025 (10)	0.0018 (10)	−0.0083 (11)
C15	0.0380 (13)	0.0415 (14)	0.0355 (12)	−0.0014 (11)	−0.0010 (10)	−0.0017 (11)
C9	0.0379 (13)	0.0454 (15)	0.0313 (12)	0.0014 (11)	0.0027 (10)	−0.0061 (11)
N3	0.0487 (12)	0.0406 (12)	0.0345 (10)	0.0035 (9)	−0.0069 (9)	−0.0001 (9)
N1	0.0385 (11)	0.0450 (12)	0.0414 (11)	0.0037 (9)	0.0025 (9)	0.0054 (10)
C13	0.0276 (11)	0.0439 (14)	0.0346 (12)	−0.0019 (10)	0.0010 (9)	0.0009 (11)
C4	0.0447 (15)	0.0487 (16)	0.0373 (13)	−0.0034 (12)	0.0056 (11)	−0.0073 (12)
C12	0.0329 (12)	0.0422 (14)	0.0331 (12)	0.0008 (10)	0.0010 (10)	0.0006 (10)
C14	0.0366 (13)	0.0502 (16)	0.0326 (12)	−0.0058 (11)	0.0007 (10)	−0.0035 (11)
C11	0.0453 (14)	0.0381 (14)	0.0415 (13)	−0.0019 (11)	0.0010 (11)	−0.0014 (11)
C1	0.0477 (16)	0.0579 (18)	0.0440 (14)	0.0072 (13)	0.0041 (12)	0.0048 (13)
C19	0.0324 (13)	0.0504 (16)	0.0574 (16)	−0.0016 (11)	0.0048 (11)	−0.0051 (13)
C21	0.0440 (14)	0.0458 (16)	0.0484 (15)	−0.0084 (12)	0.0081 (12)	−0.0052 (12)
C22	0.0588 (17)	0.0461 (16)	0.0492 (15)	−0.0045 (13)	0.0042 (13)	0.0104 (13)
C6	0.067 (2)	0.0495 (17)	0.0520 (16)	−0.0082 (14)	0.0080 (14)	0.0066 (13)
C17	0.0412 (14)	0.0404 (15)	0.0479 (14)	0.0023 (11)	0.0007 (11)	0.0053 (12)
C18	0.0353 (14)	0.0530 (17)	0.0634 (17)	0.0087 (12)	−0.0035 (12)	0.0026 (14)
C2	0.0413 (15)	0.076 (2)	0.0586 (17)	0.0156 (14)	0.0047 (13)	0.0043 (16)
C23	0.0528 (17)	0.0515 (17)	0.0507 (16)	0.0107 (13)	0.0016 (12)	0.0124 (13)
C7	0.0529 (17)	0.0523 (18)	0.0575 (17)	0.0025 (13)	0.0021 (13)	0.0093 (14)
C3	0.0374 (15)	0.072 (2)	0.0567 (17)	0.0002 (13)	0.0064 (12)	−0.0029 (15)
C5	0.0480 (16)	0.0574 (18)	0.0505 (16)	−0.0131 (13)	0.0100 (12)	−0.0044 (14)
O1W	0.0971 (18)	0.0642 (15)	0.0753 (16)	−0.0058 (13)	−0.0050 (13)	−0.0143 (13)

### Geometric parameters (Å, °)

**Table:**

S1—C12	1.750 (2)	N1—H1	0.8600
S1—C11	1.803 (3)	C4—C5	1.407 (4)
S2—C13	1.748 (2)	C4—C3	1.414 (4)
S2—C14	1.801 (2)	C14—H14A	0.9700
S3—C12	1.723 (2)	C14—H14B	0.9700
S3—C13	1.731 (2)	C11—H11A	0.9700
N2—C15	1.343 (3)	C11—H11B	0.9700
N2—C16	1.408 (3)	C1—C2	1.408 (4)
N2—H2	0.8600	C1—H1A	0.9300
N4—C13	1.299 (3)	C19—C18	1.353 (4)
N4—N3	1.392 (3)	C19—H19	0.9300
N5—C7	1.313 (3)	C21—C22	1.352 (4)
N5—C8	1.365 (3)	C21—H21	0.9300
N6—C23	1.316 (3)	C22—C23	1.403 (4)
N6—C24	1.367 (3)	C22—H22	0.9300
O2—C15	1.222 (3)	C6—C5	1.357 (4)
C24—C20	1.414 (3)	C6—C7	1.400 (4)
C24—C16	1.424 (3)	C6—H6	0.9300
C16—C17	1.370 (3)	C17—C18	1.406 (3)
O1—C10	1.221 (3)	C17—H17	0.9300
C8—C4	1.414 (3)	C18—H18	0.9300
C8—C9	1.424 (3)	C2—C3	1.347 (4)
C10—N1	1.345 (3)	C2—H2A	0.9300
C10—C11	1.517 (3)	C23—H23	0.9300
C20—C21	1.409 (3)	C7—H7	0.9300
C20—C19	1.413 (3)	C3—H3	0.9300
C15—C14	1.516 (3)	C5—H5	0.9300
C9—C1	1.367 (3)	O1W—H1WA	0.95 (6)
C9—N1	1.408 (3)	O1W—H1WB	0.93 (4)
N3—C12	1.298 (3)		
			
C12—S1—C11	99.71 (11)	C15—C14—H14B	107.6
C13—S2—C14	100.22 (11)	S2—C14—H14B	107.6
C12—S3—C13	86.72 (11)	H14A—C14—H14B	107.1
C15—N2—C16	127.9 (2)	C10—C11—S1	117.78 (17)
C15—N2—H2	116.0	C10—C11—H11A	107.9
C16—N2—H2	116.0	S1—C11—H11A	107.9
C13—N4—N3	112.00 (18)	C10—C11—H11B	107.9
C7—N5—C8	117.0 (2)	S1—C11—H11B	107.9
C23—N6—C24	117.0 (2)	H11A—C11—H11B	107.2
N6—C24—C20	122.5 (2)	C9—C1—C2	119.9 (3)
N6—C24—C16	118.1 (2)	C9—C1—H1A	120.0
C20—C24—C16	119.3 (2)	C2—C1—H1A	120.0
C17—C16—N2	124.3 (2)	C18—C19—C20	120.0 (2)
C17—C16—C24	119.8 (2)	C18—C19—H19	120.0
N2—C16—C24	115.88 (19)	C20—C19—H19	120.0
N5—C8—C4	123.0 (2)	C22—C21—C20	119.3 (2)
N5—C8—C9	118.0 (2)	C22—C21—H21	120.3
C4—C8—C9	119.1 (2)	C20—C21—H21	120.3
O1—C10—N1	124.5 (2)	C21—C22—C23	119.1 (2)
O1—C10—C11	118.9 (2)	C21—C22—H22	120.4
N1—C10—C11	116.6 (2)	C23—C22—H22	120.4
C21—C20—C19	123.2 (2)	C5—C6—C7	118.7 (3)
C21—C20—C24	117.6 (2)	C5—C6—H6	120.7
C19—C20—C24	119.2 (2)	C7—C6—H6	120.7
O2—C15—N2	123.9 (2)	C16—C17—C18	119.9 (2)
O2—C15—C14	118.1 (2)	C16—C17—H17	120.0
N2—C15—C14	117.8 (2)	C18—C17—H17	120.0
C1—C9—N1	124.6 (2)	C19—C18—C17	121.7 (2)
C1—C9—C8	120.1 (2)	C19—C18—H18	119.2
N1—C9—C8	115.3 (2)	C17—C18—H18	119.2
C12—N3—N4	112.3 (2)	C3—C2—C1	121.5 (3)
C10—N1—C9	129.6 (2)	C3—C2—H2A	119.3
C10—N1—H1	115.2	C1—C2—H2A	119.3
C9—N1—H1	115.2	N6—C23—C22	124.3 (2)
N4—C13—S3	114.42 (17)	N6—C23—H23	117.8
N4—C13—S2	126.16 (17)	C22—C23—H23	117.8
S3—C13—S2	119.42 (14)	N5—C7—C6	124.5 (3)
C5—C4—C3	123.9 (2)	N5—C7—H7	117.8
C5—C4—C8	117.0 (2)	C6—C7—H7	117.8
C3—C4—C8	119.1 (2)	C2—C3—C4	120.3 (2)
N3—C12—S3	114.56 (17)	C2—C3—H3	119.8
N3—C12—S1	125.12 (19)	C4—C3—H3	119.8
S3—C12—S1	120.31 (13)	C6—C5—C4	119.9 (2)
C15—C14—S2	118.78 (16)	C6—C5—H5	120.1
C15—C14—H14A	107.6	C4—C5—H5	120.1
S2—C14—H14A	107.6	H1WA—O1W—H1WB	99 (4)
			
C23—N6—C24—C20	2.3 (3)	N4—N3—C12—S3	0.5 (3)
C23—N6—C24—C16	−177.1 (2)	N4—N3—C12—S1	179.22 (15)
C15—N2—C16—C17	10.7 (4)	C13—S3—C12—N3	−0.37 (18)
C15—N2—C16—C24	−170.7 (2)	C13—S3—C12—S1	−179.18 (15)
N6—C24—C16—C17	179.8 (2)	C11—S1—C12—N3	−3.3 (2)
C20—C24—C16—C17	0.3 (3)	C11—S1—C12—S3	175.39 (14)
N6—C24—C16—N2	1.1 (3)	O2—C15—C14—S2	−165.45 (19)
C20—C24—C16—N2	−178.34 (19)	N2—C15—C14—S2	18.5 (3)
C7—N5—C8—C4	1.0 (3)	C13—S2—C14—C15	87.36 (19)
C7—N5—C8—C9	−178.3 (2)	O1—C10—C11—S1	154.2 (2)
N6—C24—C20—C21	−2.6 (3)	N1—C10—C11—S1	−28.2 (3)
C16—C24—C20—C21	176.8 (2)	C12—S1—C11—C10	−64.46 (19)
N6—C24—C20—C19	178.5 (2)	N1—C9—C1—C2	−178.6 (2)
C16—C24—C20—C19	−2.1 (3)	C8—C9—C1—C2	1.3 (4)
C16—N2—C15—O2	−3.4 (4)	C21—C20—C19—C18	−177.0 (2)
C16—N2—C15—C14	172.5 (2)	C24—C20—C19—C18	1.8 (4)
N5—C8—C9—C1	178.4 (2)	C19—C20—C21—C22	−180.0 (2)
C4—C8—C9—C1	−0.9 (3)	C24—C20—C21—C22	1.2 (3)
N5—C8—C9—N1	−1.7 (3)	C20—C21—C22—C23	0.4 (4)
C4—C8—C9—N1	179.0 (2)	N2—C16—C17—C18	−179.7 (2)
C13—N4—N3—C12	−0.3 (3)	C24—C16—C17—C18	1.8 (3)
O1—C10—N1—C9	−2.8 (4)	C20—C19—C18—C17	0.3 (4)
C11—C10—N1—C9	179.8 (2)	C16—C17—C18—C19	−2.1 (4)
C1—C9—N1—C10	−2.6 (4)	C9—C1—C2—C3	−0.3 (4)
C8—C9—N1—C10	177.5 (2)	C24—N6—C23—C22	−0.6 (4)
N3—N4—C13—S3	0.1 (2)	C21—C22—C23—N6	−0.7 (4)
N3—N4—C13—S2	179.95 (15)	C8—N5—C7—C6	−0.4 (4)
C12—S3—C13—N4	0.17 (18)	C5—C6—C7—N5	−0.7 (4)
C12—S3—C13—S2	−179.74 (14)	C1—C2—C3—C4	−1.1 (4)
C14—S2—C13—N4	−5.8 (2)	C5—C4—C3—C2	−177.7 (3)
C14—S2—C13—S3	174.10 (13)	C8—C4—C3—C2	1.4 (4)
N5—C8—C4—C5	−0.6 (3)	C7—C6—C5—C4	1.1 (4)
C9—C8—C4—C5	178.7 (2)	C3—C4—C5—C6	178.6 (3)
N5—C8—C4—C3	−179.8 (2)	C8—C4—C5—C6	−0.5 (4)
C9—C8—C4—C3	−0.4 (3)		

### Hydrogen-bond geometry (Å, °)

**Table:**

*D*—H···*A*	*D*—H	H···*A*	*D*···*A*	*D*—H···*A*
O1W—H1WA···O1	0.95 (7)	2.07 (7)	2.990 (3)	162 (6)
O1W—H1WB···O2^i^	0.94 (4)	1.91 (4)	2.841 (3)	175 (4)
C11—H11B···O1W^ii^	0.97	2.49	3.332 (4)	145
C23—H23···O1^iii^	0.93	2.55	3.411 (4)	155

Symmetry codes: (i) −*x*+3/2, *y*−1/2, −*z*+1/2; (ii) −*x*+1, −*y*, −*z*; (iii) *x*, *y*+1, *z*.
